# Comparative RNA-Seq Analysis of Early-Infected Peach Leaves by the Invasive Phytopathogen *Xanthomonas arboricola* pv. *pruni*

**DOI:** 10.1371/journal.pone.0054196

**Published:** 2013-01-14

**Authors:** Didier Socquet-Juglard, Tim Kamber, Joël F. Pothier, Danilo Christen, Cesare Gessler, Brion Duffy, Andrea Patocchi

**Affiliations:** 1 Phytopathology, Research Station Agroscope Changins-Wädenswil (ACW), Wädenswil, Switzerland; 2 Plant Pathology, Swiss Federal Institute of Technology (ETH Zürich), IBZ, Zürich, Switzerland; 3 Berries, Medicinal Plants, Greenhouse Crops and Apricots Group, Research Station Agroscope Changins-Wädenswil, Conthey Research Centre, Conthey, Switzerland; Naval Research Laboratory, United States of America

## Abstract

*Xanthomonas arboricola* pv. *pruni* is a quarantine bacterial pathogen that threatens peach production by causing necrotic spots on leaves and fruits, thus with the potential of severely reducing yields. The current understanding of the host plant defense responses to the pathogen is very limited. Using whole transcriptome sequencing, differential gene expression was analyzed at two time points, 2 h and 12 h post inoculation (hpi), by comparing the inoculated samples to their respective controls. On the total of 19,781 known peach genes that were expressed in all time points and conditions, 34 and 263 were differentially expressed at 2 and 12 hpi, respectively. Of those, 82% and 40% were up-regulated, respectively; and 18% and 60% were down-regulated, respectively. The functional annotation based on gene ontology (GO) analysis highlighted that genes involved in metabolic process and response to stress were particularly represented at 2 hpi whereas at 12 hpi cellular and metabolic processes were the categories with the highest number of genes differentially expressed. Of particular interest among the differentially expressed genes identified were several pathogen-associated molecular pattern (PAMP) receptors, disease resistance genes including several RPM1-like and pathogenesis related thaumatin encoding genes. Other genes involved in photosynthesis, in cell wall reorganization, in hormone signaling pathways or encoding cytochrome were also differentially expressed. In addition, novel transcripts were identified, providing another basis for further characterization of plant defense-related genes. Overall, this study gives a first insight of the peach defense mechanisms during the very early stages of infection with a bacterial disease in the case of a compatible interaction.

## Introduction

Bacterial spot of stone fruits, caused by *Xanthomonas arboricola* pv. *pruni*, is a severe disease that threaten the most economically important *Prunus* crops, including peach, apricot, nectarine and plum. Identified for the first time in 1903 in the USA [1], the disease has spread worldwide and has been now reported from all continents [2]. The pathogen uses wounds or stomata to access the intercellular spaces where it degrades the cell wall components [3]. This causes necrotic lesions on both leaves and fruits, leading to severe defoliations and yield losses. In some cases the pathogen can be also responsible for cankers and death of the trees [4]. The pathogenicity of *X. arboricola* pv. *pruni* relies on a large repertoire of 21 type III effectors (T3Es) which can be delivered directly into the host cells via the type III secretion system [5]. T3Es are known to promote bacterial growth in the host plant by suppressing plant defenses [6].

Despite extensive efforts to characterize cultivars among several *Prunus* species according to their level of resistance to *Xanthomonas arboricola* pv. *pruni* [7], [8], [9], not much is known about the genetics underlying host defense responses. Recently, one major QTL for disease incidence in apricot has been identified on linkage group 5 [10]. In peach, major QTLs for *X. arboricola* pv. *pruni* resistance have also been recently identified, one on LG4 for leaf resistance, one on LG5 for both fruit and leaf resistance, and one on each LG1 and LG6 for fruit resistance [11]. In addition, the differential expression of pathogen-related genes in peach identified by qRT-PCR upon bacterial spot infection, as well as after methyl jasmonate and ethephon treatments, showed that jasmonic acid and ethylene pathways may play a role in disease resistance [12].

*X. arboricola* pv. *pruni* is especially virulent on peach [*Prunus persica* (L.) Batsch], which is one of the most economically important species in the genus *Prunus,* in terms of tonnage and production area [13]. Characterized by eight chromosomes (2n = 16) and a small genome size (around 227 Mbp) in comparison to other plant species [14], peach is considered as a model species in the Rosaceae family [15], [16]. As a consequence, a great emphasis has been placed on developing efficient marker-assisted selection strategies to fasten molecular breeding [17]. Several inter- and intraspecific genetic linkage maps have been constructed; one of those, ‘Texas’ (almond) × ‘Earlygold’ (peach) has been saturated with markers and is considered as the reference map for *Prunus* [18], [19]. From these maps, Quantitative Trait Loci (QTLs) involved in fruit quality, adaptation, and disease resistance have been identified [20]. Major recent advances have been the release of the complete peach genome sequence by the IPGI (International Peach Genome Initiative, [21], [22]) and the development of an Illumina 9,000 SNP array by the IPSC (International Peach SNP Consortium, [23]) which permit to efficiently improve coverage and saturation of linkage maps [11], [24].

Microarrays have been extensively used in the past years to study the expression levels of transcripts in many plants including *Prunus* species [25], [26], [27], [28], [29]. It notably permitted to show in *Arabidopsis thaliana* that the same set of genes confers resistance or susceptibility to diseases, and that the difference of phenotype is due to the timing and magnitude of the expression of those genes [30]. However, microarray technology presents drawbacks including a limitation to known transcripts and background signals leading to low sensitivities for low expressed genes. These limitations have been overcome with recent advances of next-generation sequencing technologies such as RNA-seq [31]. RNA-seq technology has become more affordable in the recent years, especially in the case of the analysis of a limited number of samples. This technology is very powerful for the analysis of transcriptomes due to the precise measure of the expression level of each gene in a sample by mapping short cDNA sequences (reads) on a reference genome. Next-generation technologies, especially after the development of the Illumina Genome Analyzer, have been successfully used to investigate differential gene expression in several pathosystems, like *Xanthomonas axonopodis* pv. *glycines* in soybean [32], *Sclerotinia homoeocarpa* in creeping bentgrass [33], or *Pseudoperonospora cubensis* in cucumber [34].

In this study, deep RNA sequencing technology was used to analyze the transcriptome of leaves of a moderately susceptible peach cultivar [9] after *X. arboricola* pv. *pruni* inoculation and after mock-inoculation at two different time points (2 h and 12 h post inoculation, hpi). Reads obtained were mapped on the peach genome and their abundance was calculated in order to identify by pair-wise comparison genes differentially expressed in early infected leaves. We further identified and characterized novel transcripts with differential abundance levels, which could also play a role in the defense mechanisms of peach against the pathogen.

## Materials and Methods

### Biological material and inoculation procedure

The strain of *X. arboricola* pv. *pruni* CFBP 5530 for which genome was recently sequenced [35], and the moderately susceptible peach rootstock ‘GF305’ [9] were used in this study. Two-year-old peach trees (Pépinières de Saxon, Switzerland) in 5 l pots containing a mixture of peat and loam were grown for one month before inoculation under quarantine greenhouse conditions. Plants were maintained at 23°C and 80% relative humidity for 24 h before and during the inoculation process, with no additional light throughout the experiment.

Preparation of the inoculum was performed as previously described [9]. Twenty fully developed leaves per tree were randomly inoculated with a needless syringe at eight different points on the abaxial side of the leaf until a water-soaked spot was clearly visible. Controls were inoculated using the same procedure but with a sterile 0.8% KCl solution, the same solution used to suspend the bacteria. Three trees (biological replicates) per time point and per type of inoculum were used. Leaf area around inoculation corresponding to the water-soak spots were collected at 2 and 12 hpi, flash frozen in liquid nitrogen and stored at −80°C until RNA extraction.

### RNA extraction and sequencing

Total RNA was isolated separately from approximately 150 mg of leaf tissue from each tree using the protocol developed by Schenk and colleagues [36] with the following modification: grinded tissue was incubated for 10 min at 65°C instead of 90 s at 95°C in the BPEX extraction buffer. RNA samples were treated with RNAse-free DNAse I (Fermentas, Switzerland) to remove contaminating DNA. To ensure that all genomic DNA was digested, samples were checked with a multiplex PCR using the 4 SSR markers UDAp-416, UDAp-487, AMPA105 and UDAp-424 using the protocol as described in [10]. Purity and concentration of the samples were estimated with a NanoDrop ND-1000 spectrophotometer (NanoDrop technologies Inc, USA) and the integrity of the RNA was evaluated on an RNA 6000 Nano LabChip using Agilent 2100 Bioanalyzer (Agilent Technologies, Germany). The three biological repetitions per treatment were then diluted at equal concentrations and equal amounts were pooled to obtain a final quantity of 5 µg RNA per condition. Four indexed strand-specific cDNA libraries were prepared and samples were sequenced on an Illumina HiSeq 2000 with a 51-bp single-end read length (GATC Biotech, Germany).

### Reads mapping and annotations

Reads were mapped to the *P. persica* genome v.1.0 obtained from the Genome Database for Rosaceae (GDR, http://www.rosaceae.org/peach/genome) using TopHat v.2.0.3 [37], with Bowtie v.0.12.8 [38].

Transcript abundance and differential gene expression were calculated with the program Cufflinks v.2.0.1 [39]. Annotations of differentially expressed genes including Pfam database [40] were obtained from the reference annotation of the peach genome available at the GDR website. Gene expression levels were normalized using fragments per kilobase of exon per million mapped reads (FPKM) report values. Genes were considered as induced or repressed, only when their log_2_ fold change was >2 or <−2, respectively, and their *P* value was <0.001.

For each differentially expressed peach gene, latest Gene Ontology (GO) annotations were obtained using Blast2GO v.2.3.5 [41] with the default parameters, and GOslim option was set to reduce the number of functional classes. GOslim annotations results were then used as queries against the AgBase database [42] to perform their classification according to the three main classes (molecular function, biological process and cellular component), and to be further assigned to secondary categories.

## Results and Discussion

### Analysis of RNA-Seq datasets

Sequencing of cDNA samples yielded 49 to 54.7 million reads corresponding to over 2.5 billion nucleotides of cDNA per sample (Table 1). Good quality scores of the reads were obtained, with Q20 percentages (sequencing error rate lower than 1%) which were all over 97%, while N percentages were all around 0.01% (Table 1). Between 73.8 and 76.9% of the reads could be mapped on the peach genome whereas an insignificant number of reads (less than 0.03% of the reads) were mapped to the *X. arboricola* pv. *pruni* CFBP 5530 genome. However, since reads from the mock-inoculated samples also mapped on *X. arboricola* pv. *pruni* genome to the same extent, this observation could be due to short identical sequences shared between the two genomes. This hypothesis is further supported by the fact that reads obtained from *X. arboricola* pv. *pruni* from *in vitro* culture mapped to the *P. persica* genome (our unpublished data). Thus we can consider that mapped reads were almost exclusively constituted of peach reads. Total number of expressed genes was over 21,000 per sample (Table 1), and a total number of 19,781 expressed genes were in common to all experiment time points and conditions (data not shown). Fragments per kilobase of exon per million mapped reads (FPKM) values of the four sequenced samples ranged from 0 for all samples to 23,273 for the 2 hpi inoculated sample (Table 1). Subsets of 5 to 40 million reads were randomly selected from the total pool of reads of each sample at each time point in order to check the effect of sampling depth on gene expression. The simulation obtained revealed that the number of genes expressed started to reach a plateau at 30 million reads, showing that the depth used in our study was sufficient to cover the whole peach transcriptome (Fig. S1).

**Table 1:** Statistics of the reads obtained and their mapping on the peach and *X. arboricola* pv. *pruni* genomes.

		Total reads	Totalnucleotides	Mapped reads^1^	% *Pp* 2	% *Xap* 3	GC content (%)	Q20 score (%)^4^	% of N^5^	Expressedgenes	Min FPKM value	Max FPKM value
Control	2 h	50,048,941	2,552,495,991	38,434,186	76.97	0.01	44.7	97.51	0.01	21,096	0	19817
	12 h	52,975,371	2,701,743,921	39,036,493	73.82	0.03	44.5	97.60	0.01	21,650	0	19971
Inoculated	2 h	49,049,564	2,501,527,764	37,223,827	76.02	0.03	44.6	97.61	0.01	21,260	0	23273
	12 h	54,747,928	2,792,144,328	41,124,163	75.38	0.01	44.0	97.60	0.01	21,550	0	16028

1 Total number of reads mapped on the *P. persica* genome.

2 Percentage of reads mapped on the *P. persica* genome.

3 Percentage of reads mapped on the *X. arboricola* pv. *pruni* CFBP 5530 genome.

4 Percentage of reads with average Phred quality score equal or above 20 i.e. for which the percentage of bases for which the accuracy of base calling is 99% or higher.

5 Percentage of nucleotides that could not be sequenced.

To evaluate gene expression, ten housekeeping genes coding for actin, tubulin, catalase or GAPDH motives (Table 2) were selected based on the study from Cocker and Davies [43]. Based on pair-wise comparisons of the inoculated samples and their respective mock-inoculated samples at the two time points, none of these reference genes was significantly differentially expressed, with log_2_ fold changes ranging from −1.05 for gene ppa005765 coding for tubulin at 12 hpi to 0.41 for another tubulin encoding gene (ppa005785) at 12 hpi. These results indicated that the sequences obtained and the transcript expression levels met the requirements for further transcriptome analysis.

**Table 2:** Expression levels of selected peach housekeeping genes.

Gene locus tag	Conserved domain	FPKM C2	FPKM I2	log2 (fold_change)	*P* value	q value	Significant	FPKM C12	FPKM I12	log2 (fold_change)	*P* value	q value	Significant
ppa005785m	tubulin	12.56	13.70	0.13	0.57	0.89	no	9.42	12.52	0.41	0.23	0.62	no
ppa005765m	tubulin	3.05	1.94	−0.65	0.03	0.27	no	7.42	3.58	−1.05	0.01	0.13	no
ppa007242m	actin	244.22	257.00	0.07	0.75	0.94	no	321.06	359.98	0.17	0.70	0.89	no
ppa005698m	tubulin	8.70	10.41	0.26	0.25	0.69	no	15.32	8.89	−0.78	0.02	0.18	no
ppa004851m	tubulin	21.13	24.55	0.22	0.31	0.74	no	36.36	30.01	−0.28	0.36	0.72	no
ppa006087m	GAPDH	66.09	64.60	−0.03	0.88	0.98	no	102.31	173.77	0.76	0.02	0.21	no
ppa008250m	GAPDH	401.98	406.15	0.01	0.95	0.99	no	299.60	264.03	−0.18	0.63	0.86	no
ppa022368m	GAPDH	0.03	0.02	−0.94	0.77	1.00	no	0.07	0.06	−0.38	0.82	1.00	no
ppa004763m	catalase	837.87	782.00	−0.10	0.70	0.93	no	2060.39	1922.25	−0.10	0.92	0.97	no
ppa004776m	catalase	1579.54	1374.63	−0.20	0.47	0.84	no	1281.23	1032.53	−0.31	0.67	0.87	no

C2 and C12 are the FPKM values obtained for the mock-inoculated samples at 2 hpi and 12 hpi, respectively. I2 and I12 are the FPKM values for the inoculated samples at 2 hpi and 12 hpi, respectively.

### Response to *X. arboricola* pv. *pruni* inoculation at 2 h and 12 h post inoculation

By performing a pair-wise comparison of the inoculated and the mock-inoculated samples at 2 hpi, 34 genes were differentially expressed due to inoculation with the bacteria; 18% of them were down-regulated and 82% were up-regulated (Table S1). The pair-wise comparison of the inoculated and mock-inoculated samples at 12 hpi resulted in the identification of a total of 263 genes differentially expressed, 60% and 40% being down-regulated and up-regulated, respectively (Table S2). The high number of down-regulated genes at 12 hpi compared to 2 hpi may reflect the release of type III effectors by the bacterial cells to suppress plant defense pathways. Four genes were in common between the two time points. One of them was up-regulated in both cases while the expression of the three others changed between 2 and 12 hpi, with two up-regulated genes at 2 hpi which were down-regulated at 12 hpi, and one gene the opposite (Table S3).

### Identification of differentially expressed potential novel genes

One advantage of the RNA-seq technology is that a part of the reads obtained may be mapped in regions of the genome of the organism under study which have not yet been annotated, thus identifying new coding regions. Here, a total of 28 and 199 novel transcripts with differential abundance levels (referred hereafter as set of novel transcripts) were identified 2 and 12 hpi, respectively, with seven being common to both time points (Table S4, Table S5, Table S6). At 2 hpi, 32% of the novel transcripts were significantly more abundant in the inoculated sample than in the control, while at 12 hpi 81% were significantly more abundant in the control sample. Using Blast2GO software, 21% and 43% of these novel transcripts could be annotated at 2 hpi and 12 hpi, respectively (Table S4, S5).

### Gene ontology analysis

**Figure 1:**
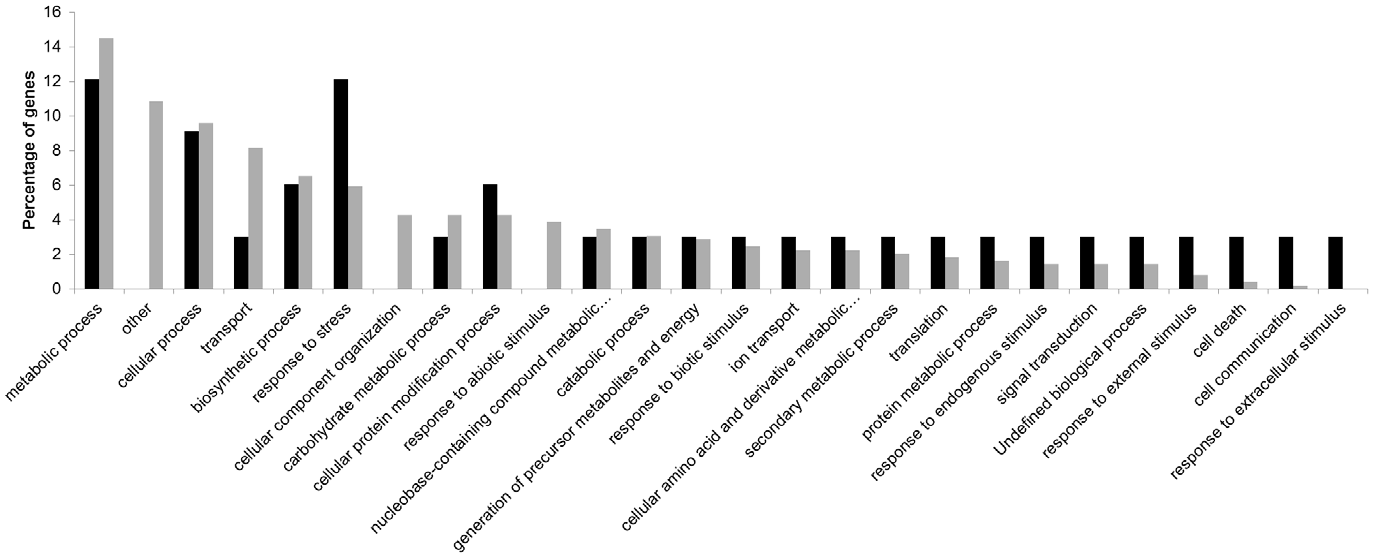
Distribution of the differentially expressed genes within the GO secondary categories of biological processes at 2 **h (in black) and 12** **h (in grey) post inoculation (hpi).**

**Figure 2:**
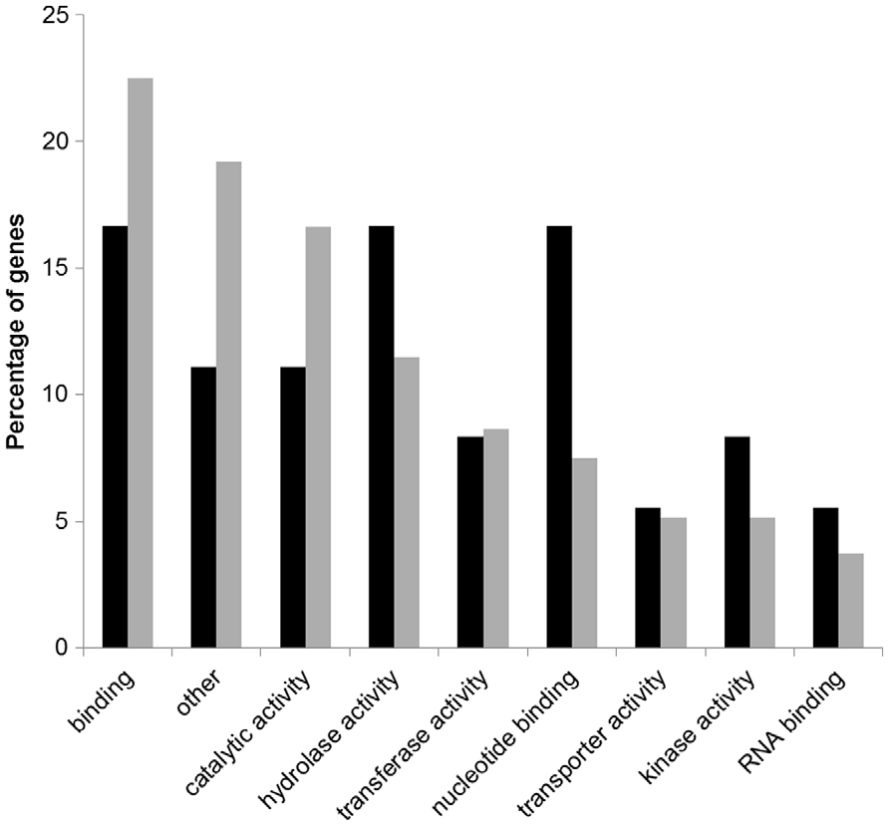
Distribution of the differentially expressed genes within the GO secondary categories of molecular functions at 2 **h (in black) and 12** **h (in grey) post inoculation (hpi).**

**Figure 3:**
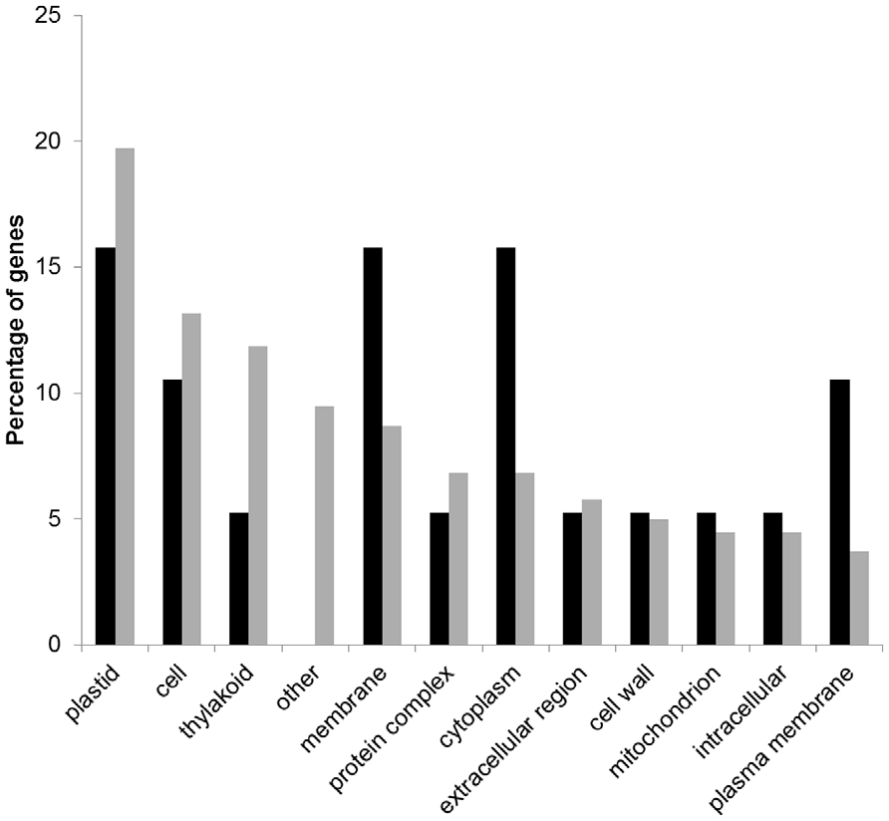
Distribution of the differentially expressed genes within the GO secondary categories of cellular components at 2 **h (in black) and 12** **h (in grey) post inoculation (hpi).**

For a better understanding of the range of genes involved in the response of peach to the inoculation of *X. arboricola* pv. *pruni*, functional classes of differentially expressed genes were determined using gene ontology (GO) analysis. Blast2GO software returned functions for 43% and 59% of the differentially expressed genes and novel transcripts at 2 and 12 hpi, respectively (Table 3). Generally for both time points, more genes were assigned to the biological process and molecular function categories than in the cellular component category (Table 4, Table 5, Table 6). The distribution of the GO functions revealed that metabolic process (12.1%) and response to stress (12.1%) were the most represented secondary categories of the biological processes at 2 hpi which may reflect that the defense mechanisms of the peach plants were activated by the pathogen already at 2 hpi (Fig. 1), while at 12 hpi the most represented biological processes identified were the metabolic (14.5%) and cellular (9.6%) processes (Fig. 1). In the category of molecular functions, a higher proportion of genes for which products were involved in binding, kinase activity, hydrolases and transferases was identified at both 2 and 12 hpi (Fig. 2). An important number of cellular component GO terms was associated with plastids, membranes, thylakoids and cell components at both 2 and 12 hpi (Fig. 3).

**Table 3:** BLASTP statistics of the differentially expressed genes at 2 and 12 hpi according to GO and Pfam databases, and similarities between peach and *Arabidopsis thaliana* transcripts.

	2 hpi		12 hpi	
	Num.	%	Num.	%
Known GO^a^	27	43.55	274	59.31
Unknown GO^a^	35	56.45	188	40.69
Known Pfam^b^	26	76.47	209	79.47
Unknown Pfam^b^	8	23.53	54	20.53
*At* 1 sequence^b^	33	97.06	250	95.06
No *At* 2 sequence^b^	1	2.94	13	4.94

a Annotation results were obtained from Blast2GO software and includes the novel transcripts.

b Annotation were obtained from the GDR annotations and does not include the novel transcripts.

1 Number and percentage of genes in peach with a corresponding transcript in the *Arabidopsis thaliana* genome.

2 Number and percentage of genes in peach without a corresponding transcript in the *A. thaliana* genome.

**Table 4:** GO functional categorization of differentially expressed peach genes with a biological process after *X. arboricola* pv. *pruni* inoculation.

		2 h				12 h				
		Up-regulated		Down-regulated		Up-regulated		Down-regulated		
GO ID	Biological process	Num	(%)	Num	(%)	Num	(%)	Num	(%)	
GO:0008152	metabolic process	3	10.34	1	25.00	20	15.87	51	14.05	
GO:0006810	transport	1	3.45	0	0.00	6	4.76	34	9.37	
GO:0009987	cellular process	1	3.45	2	50.00	16	12.70	31	8.54	
GO:0009058	biosynthetic process	2	6.90	0	0.00	5	3.97	27	7.44	
GO:0006950	response to stress	3	10.34	1	25.00	8	6.35	21	5.79	
GO:0016043	cellular component organization	0	0.00	0	0.00	5	3.97	16	4.41	
GO:0009628	response to abiotic stimulus	0	0.00	0	0.00	3	2.38	16	4.41	
GO:0006091	generation of precursor metabolites and energy	1	3.45	0	0.00	0	0.00	14	3.86	
GO:0005975	carbohydrate metabolic process	1	3.45	0	0.00	7	5.56	14	3.86	
GO:0009056	catabolic process	1	3.45	0	0.00	2	1.59	13	3.58	
GO:0006464	cellular protein modification process	2	6.90	0	0.00	9	7.14	12	3.31	
GO:0006139	nucleobase-containing compound metabolic process	1	3.45	0	0.00	5	3.97	12	3.31	
GO:0006811	ion transport	1	3.45	0	0.00	0	0.00	11	3.03	
GO:0009607	response to biotic stimulus	1	3.45	0	0.00	2	1.59	10	2.75	
GO:0006629	lipid metabolic process	0	0.00	0	0.00	4	3.17	10	2.75	
GO:0006519	cellular amino acid and derivative metabolic process	1	3.45	0	0.00	2	1.59	9	2.48	
GO:0006412	translation	1	3.45	0	0.00	0	0.00	9	2.48	
GO:0019538	protein metabolic process	1	3.45	0	0.00	1	0.79	7	1.93	
GO:0019748	secondary metabolic process	1	3.45	0	0.00	3	2.38	7	1.93	
GO:0007275	multicellular organismal development	0	0.00	0	0.00	1	0.79	7	1.93	
GO:0015979	photosynthesis	0	0.00	0	0.00	0	0.00	5	1.38	
GO:0009719	response to endogenous stimulus	1	3.45	0	0.00	3	2.38	4	1.10	
GO:0009653	anatomical structure morphogenesis	0	0.00	0	0.00	1	0.79	4	1.10	
GO:0030154	cell differentiation	0	0.00	0	0.00	0	0.00	4	1.10	
GO:0007165	signal transduction	1	3.45	0	0.00	4	3.17	3	0.83	
GO:0009791	post-embryonic development	0	0.00	0	0.00	2	1.59	3	0.83	
GO:0016049	cell growth	0	0.00	0	0.00	2	1.59	2	0.55	
GO:0008150	undefined biological process	1	3.45	0	0.00	6	4.76	1	0.28	
GO:0009605	response to external stimulus	1	3.45	0	0.00	3	2.38	1	0.28	
GO:0000003	reproduction	0	0.00	0	0.00	1	0.79	1	0.28	
GO:0007049	cell cycle	0	0.00	0	0.00	0	0.00	1	0.28	
GO:0007610	behavior	0	0.00	0	0.00	0	0.00	1	0.28	
GO:0009908	flower development	0	0.00	0	0.00	0	0.00	1	0.28	
GO:0040007	growth	0	0.00	0	0.00	0	0.00	1	0.28	
GO:0007154	cell communication	1	3.45	0	0.00	1	0.79	0	0.00	
GO:0008219	cell death	1	3.45	0	0.00	2	1.59	0	0.00	
GO:0009991	response to extracellular stimulus	1	3.45	0	0.00	0	0.00	0	0.00	
GO:0008037	cell recognition	0	0.00	0	0.00	1	0.79	0	0.00	
GO:0006259	DNA metabolic process	0	0.00	0	0.00	1	0.79	0	0.00	
Total		29	100	4	100	126	100	363	100	

**Table 5:** GO functional categorization of differentially expressed peach genes with a molecular function after *X. arboricola* pv. *pruni* inoculation.

		2 h				12 h			
		Up-regulated		Down-regulated		Up-regulated		Down-regulated	
GO ID	Molecular function	Num	(%)	Num	(%)	Num	(%)	Num	(%)
GO:0005488	binding	6	20.00	0	0.00	18	14.63	78	25.66
GO:0003824	catalytic activity	2	6.67	2	33.33	14	11.38	57	18.75
GO:0016787	hydrolase activity	5	16.67	1	16.67	14	11.38	35	11.51
GO:0016740	transferase activity	3	10.00	0	0.00	16	13.01	21	6.91
GO:0005215	transporter activity	2	6.67	0	0.00	3	2.44	19	6.25
GO:0009055	electron carrier activity	0	0.00	0	0.00	0	0.00	18	5.92
GO:0000166	nucleotide binding	5	16.67	1	16.67	15	12.20	17	5.59
GO:0005515	protein binding	1	3.33	0	0.00	5	4.07	14	4.61
GO:0003723	RNA binding	2	6.67	0	0.00	4	3.25	12	3.95
GO:0005198	structural molecule activity	1	3.33	0	0.00	0	0.00	10	3.29
GO:0016301	kinase activity	2	6.67	1	16.67	17	13.82	5	1.64
GO:0030234	enzyme regulator activity	0	0.00	0	0.00	0	0.00	3	0.99
GO:0030246	carbohydrate binding	0	0.00	0	0.00	1	0.81	3	0.99
GO:0003677	DNA binding	0	0.00	1	16.67	5	4.07	2	0.66
GO:0003700	sequence-specific DNA binding transcription factor activity	0	0.00	0	0.00	2	1.63	2	0.66
GO:0008289	lipid binding	0	0.00	0	0.00	0	0.00	2	0.66
GO:0019825	oxygen binding	0	0.00	0	0.00	0	0.00	2	0.66
GO:0003676	nucleic acid binding	0	0.00	0	0.00	4	3.25	1	0.33
GO:0004518	nuclease activity	1	3.33	0	0.00	0	0.00	1	0.33
GO:0004872	receptor activity	0	0.00	0	0.00	2	1.63	1	0.33
GO:0008135	translation factor activity, nucleic acid binding	0	0.00	0	0.00	0	0.00	1	0.33
GO:0004871	signal transducer activity	0	0.00	0	0.00	3	2.44	0	0.00
Total		30	100	6	100	123	100	304	100

**Table 6:** GO functional categorization of differentially expressed peach genes involved in cellular components after *X. arboricola* pv. *pruni* inoculation.

		2 h				12 h				
		Up-regulated		Down-regulated		Up-regulated		Down-regulated		
GO ID	Cellular component	Num	(%)	Num	(%)	Num	(%)	Num	(%)	
GO:0009536	plastid	3	16.67	0	0.00	4	9.30	71	21.07	
GO:0005623	cell	1	5.56	1	100.00	5	11.63	45	13.35	
GO:0009579	thylakoid	1	5.56	0	0.00	0	0.00	45	13.35	
GO:0016020	membrane	3	16.67	0	0.00	5	11.63	28	8.31	
GO:0043234	protein complex	1	5.56		0.00	0	0.00	26	7.72	
GO:0005737	cytoplasm	3	16.67	0	0.00	3	6.98	23	6.82	
GO:0005739	mitochondrion	1	5.56	0	0.00	0	0.00	17	5.04	
GO:0005576	extracellular region	1	5.56	0	0.00	7	16.28	15	4.45	
GO:0005618	cell wall	1	5.56	0	0.00	4	9.30	15	4.45	
GO:0005622	intracellular	1	5.56	0	0.00	3	6.98	14	4.15	
GO:0005634	nucleus	0	0.00	0	0.00	4	9.30	9	2.67	
GO:0005829	cytosol	0	0.00	0	0.00	1	2.33	9	2.67	
GO:0005886	plasma membrane	2	11.11	0	0.00	7	16.28	7	2.08	
GO:0005773	vacuole	0	0.00	0	0.00	0	0.00	5	1.48	
GO:0005777	peroxisome	0	0.00	0	0.00	0	0.00	3	0.89	
GO:0005783	endoplasmic reticulum	0	0.00	0	0.00	0	0.00	2	0.59	
GO:0005730	nucleolus	0	0.00	0	0.00	0	0.00	1	0.30	
GO:0005794	Golgi apparatus	0	0.00	0	0.00	0	0.00	1	0.30	
GO:0005856	cytoskeleton	0	0.00	0	0.00	0	0.00	1	0.30	
Total		18	100	1	100	43	100	337	100	

Furthermore, GO analysis categorized up-regulated genes in 45 and 54 different functions at 2 and 12 hpi, respectively, and in 9 and 74 different functions at 2 and 12 hpi, respectively for the down-regulated genes (Table 4, Table 5 and Table 6).

### Transcriptional changes of defense-related genes using *Prunus persica* annotations

After pair-wise comparisons of inoculated samples to their respective mock-inoculated samples at 2 and 12 hpi, annotations of differentially expressed genes were obtained from the peach genome annotation. Almost all of them (97% at 2 hpi and 95% at 12 hpi) were identified with orthologs in *A. thaliana* (Table 3). Most of the genes were annotated, with 76% at 2 hpi and 79% of the genes at 12 hpi being assigned to a Pfam category (Table 3, Table S1, and Table S2).

### Genes involved in basal defense

Plants possess different sophisticated systems to defend themselves against pathogen attacks. In some cases their cells express receptors with a broad range specificity and detect structures of the pathogen called pathogen associated molecular patterns (PAMPs) leading to a PAMP-triggered immunity [44]. One of the best-characterized PAMP-triggered immunity genes is FLAGELLIN SENSING 2 (*FLS2*), a transmembrane receptor kinase for bacterial flagellin in *A. thaliana* which contains a leucin-rich (LRR) repeat domain [45]. This gene belongs to a family of receptor-like kinases including rice gene *Xa21* (*Xanthomonas* resistance protein 21, [46], [47]. In the transcriptome of soybean inoculated with *X. axonopodis* pv. *glycines,* a close homologue of *FLS2* was shown to be up-regulated at 0 hpi in a resistant line in comparison to the susceptible one but was not differentially expressed 6 and 12 hpi [32]. In our study, ppa1027223m similar to *FLS2* was up-regulated at 12 hpi in comparison to the controls (Table S2); although the expression of this gene has not been reported yet in *Prunus*, we can hypothesize that it may have a similar function as in *A. thaliana*. Two other genes with similarities to genes coding for germin proteins were identified, one (ppa016616m) was significantly up-regulated and the other (ppa011460m) down-regulated. Similar genes have been previously identified for being involved in basal plant defense against several pathogens, by playing a role in the synthesis of active oxygen [48] and were reported to be up-regulated in Italian ryegrass (*Lolium multiflorum* Lam.) upon inoculation with *X. translucens* pv. *graminis* [49].

After the perception of PAMPs, genes involved in the signaling cascade (mitogen-activated protein kinases, MAPK) are activated and followed by the activation of WRKY transcription factors [50]. Gene ppa006485m, similar to a gene encoding a mitogen-activated protein kinase kinase kinase (MAPKKK15) was identified in our study and was down-regulated while genes ppa015973m which could belong to the MYB-family and ppa018075m to the WRKY-family were both up-regulated at 12 hpi in our study (Table S2). Other homologs to genes encoding DNA binding proteins, transcriptional regulation or transcription factors were differentially expressed, such as one encoding a transducin/WD40-repeat containing protein (ppa026854m) and one belonging to the basic-helix loop helix DNA-binding family (ppa017640m) at 2 hpi. At 12 hpi, two differentially expressed genes (ppb012603m and ppa022385m) similar to genes belonging to the basic-helix loop helix DNA-binding family and four (ppa012687m, ppa012737m, ppa012242m, and ppa011359m) to genes belonging to zinc finger families were identified.

### Genes involved in cell wall reorganization

In this study, several putative genes involved in cell wall formation and degradation were identified. Ppa003528m was one of the most highly down-regulated genes in our study at 12 hpi (log_2_ fold change = −5.9, Table S2). Ppa003528m is similar to genes belonging to the invertase/pectin methylesterase inhibitor family, from which some genes are known to be involved in cell wall modification, but have also been reported as playing an important role in basal disease resistance, and can be induced by *X. campestris* pv. *vesicatoria* in pepper [51]. Invertase/pectin methylesterase inhibitors have also been shown to be down-regulated in susceptible potato plants 0.5 hours after *Potato virus Y* inoculation [52].

Seven differentially expressed genes similar to β-glucosidase encoding genes were identified at 12 hpi, one of them (ppa006110m) being the most up-regulated (log_2_ fold change = 5.9) whereas all others were down-regulated (Table S2). In plants, β-glucosidases are known to play an important role in cell wall lignification [53], but also for their activation of phytohormones [54] and chemical defense compounds [55]. Other genes which could play a role in cell wall reorganization were identified and were up-regulated, such as two xyloglucan endotransglycosylase/hydrolase (ppa019741m and ppa009608m), and one xyloglucan:xyloglucosyl transferase (ppa009090m) which have a role in primary cell wall restructuration [56]; as well as one peroxidase (ppa027053m) which can be involved in cell wall lignification and degradation of vascular tissues. Peroxidases are also known to play a role in systemic resistance of tobacco to blue mold [57] and to wounding in northern red oak [58]. Another differentially expressed gene similar to a gene encoding a cell wall hydrolase was found on scaffold 3 in the set of novel transcripts at 12 hpi and was down-regulated (Table S5). The identification of differentially expressed genes that could be involved in cell wall reorganization indicates that major changes may be performed at an early stage, either by degrading or lignifying cell walls to avoid the spread of the disease into the vascular tissues.

### Differential expression of photosynthesis genes

Five genes putatively involved in light harvesting complexes (ppa009686m and ppa004865m), or related to chlorophyll A/B binding protein (ppa010015m and ppa010034m) or to the photosystem I (ppa013313m) were all down-regulated at 12 hpi. Additionally, 14 differentially expressed genes in the set of novel transcripts were similar to genes involved in photosynthesis (photosystem I and II proteins, Table S5) and were all identified as significantly down-regulated at 12 hpi. These results are in accordance with previous observations, e.g. in the *A. thaliana – P. syringae* pathosystem [59], but also in kumquat leaves challenged by *X. citri* subsp. *citri* [60]. It was hypothesized that the down-regulation of genes involved in photosynthesis could induce a hypersensitive response following the infection [60]. This could also be due to a strategy of the plant to limit the availability of sugars for the pathogen, to fitness costs for the plant which has to reallocate for defense [61], or to protect the photosynthetic apparatus against oxidative damage [62].

### Genes involved in hormone signaling pathways

Hormones play an important role as signaling molecules in response to biotic stresses, especially salicylic acid (SA), jasmonates (JA) and ethylene (ET) (reviewed in [63]). Kim and colleagues [32] did not observe any significant differentially expressed SA-related gene in soybean after *X. axonopodis* pv. *glycines* inoculation, but identified differentially expressed JA-signaling genes. Here, no gene involved in SA-, JA- or ET-signaling pathways was differentially expressed. However, ppa004865m which is similar to *LAX2* could be involved in auxin transport, and ppa013981m similar to genes belonging to the SAUR-like auxin-responsive protein family could play a role in auxin signaling pathway [64]; both were down-regulated in our study (Table S2). Blocking auxin responses in *A. thaliana* was shown to increase plant resistance to *Pseudomonas syringae* pv. *maculicola* [65]. Furthermore, the suppression of auxin signaling pathway promotes the SA- and JA-signaling pathways, and inhibits the expansin expression in rice after *X. oryzae* pv. *oryzae* inoculation [66]. Three expansin-like genes were identified as down-regulated 12 hpi. Expansins are known as regulators of cell wall extension during cell growth. On the other side, one of the most down-regulated genes (ppa020764m, with a log_2_ fold change of −6.0, Table S2) at 12 hpi could be involved in the gibberellin-signaling pathway. Although they have been much less studied than the other defense-related signaling molecules SA, JA and ET, gibberellins could also actively play a role in the plant defense response by activating or repressing these signaling molecules [63].

### An important down-regulation of cytochrome-like genes

Among the differentially expressed genes, a very high number of down-regulated (ten down-regulated on a total of 11 differentially expressed genes, Table S2) homologs to genes encoding cytochrome P450s (CYP) were observed at 12 hpi. CYP play diverse roles such as antioxidants, UV protectants, detoxification of pollutants, biosynthesis of hormones, but are also known to be involved in basal plant defense against a wide variety of pathogens, including bacterial pathogens [67]. Another set of 13 cytochrome related genes were all down-regulated among the set of novel transcripts (Table S5). In our study, the down-regulation of CYP genes is probably one of the most pronounced transcriptional change following the infection by *X. arboricola* pv. *pruni*. This is an indication of a possible strategy for the pathogen to delay the stress signaling pathways of the plant.

### Putative resistance genes

PAMP-triggered immunity can be inactivated by bacterial type III effectors which are directly delivered into the host cells via the type III secretion system. These effectors interfere with the host cell function to induce disease [68]. They can be recognized in the plant by resistance (R) genes: this is a gene-for-gene resistance which leads to extracellular oxidative burst, induction of salicylic acid signaling pathway, calcium and hydrogen influxes in the cell, and cell death or hypersensitive response [69]. Although only a few genes were differentially expressed at 2 hpi, several putative R genes were identified, among them the two RPM1-like genes containing NB-ARC domains (ppa019283m and ppa016517m) and known for conferring resistance to *P. syringae* in *A. thaliana* [70] were up-regulated. Two others, one putative leucine-rich repeat (LRR) kinase (ppa025198m) and one coding for a putative disease resistance protein containing TIR-NBS-LRR motives (ppa024010m) were down-regulated. Among the 28 novel transcripts identified at 2 hpi, one down-regulated gene coding for a putative receptor-like kinase was detected on scaffold 2 (Table S4).

Twelve hours post inoculation, several putative R genes were differentially expressed, among them four RPM1-like genes (ppa000961m, ppa021560m, ppa024705m, ppa026627m), two putative genes coding for LRR protein kinases (ppa018586m and ppa017496m), one for cysteine proteinase (ppa008267m) that could be involved in cell death, and seven belonged to disease resistance protein families with LRR motives (ppa000999m, ppb021252m, ppa021712m), CC-NBS-LRR motives (ppa017163m, ppa001498m) or TIR-NBS-LRR motives (ppa025848m, ppa026531m). All these genes were up-regulated at 12 hpi, with log_2_ fold changes ranging from 2.0 to 3.3 (Table S2).

### Defense-related genes

Two pathogenesis related thaumatin genes (ppa010418m and ppa010410m) identified in our study were down-regulated at 12 hpi. Similar genes were reported to be overexpressed during the interaction of *X. oryzae* pv. *oryzae* with rice, and conferred a moderate level of resistance [71]. Other putative defense-related genes, such as one coding for a chitinase A (ppa026927m), five for terpene synthases (ppa016292m, ppa020831m, ppa023341m, ppa024025m, and ppa024760m) and five for glutathione S-transferases (ppa014555m, ppa018201m, ppa019045m, ppa023395m, and ppb009348m) were differentially expressed at 12 hpi. Two additional up-regulated putative genes coding for a LRR receptor-like serine threonine-protein kinase and a receptor protein kinase-like were identified among the set of novel transcripts on scaffold 6 (Table S5).

### Defense mechanisms in *Prunus* in response to *X. arboricola* pv. *pruni*

Up to now not much was known about the mechanisms of resistance against *X. arboricola* pv. *pruni* in *Prunus* species. In peach, major QTLs were associated with bacterial spot resistance on LG1, 4, 5, and 6 [11]; and in apricot, a QTL was previously mapped on linkage group 5 of ‘Rouge de Mauves’ with data issued from evaluations of two consecutive years [10]. Apricot and peach genomes being highly syntenic [72], [73], the scaffold 5 of peach corresponds to the linkage group 5 of apricot. Thus we searched for differentially expressed genes located on this scaffold. Four and 17 differentially expressed genes on scaffold 5 were identified at 2 h and 12 hpi, respectively. However, only one (ppa011598m, Table S5) was in the confidence interval of the mapped QTL [10] and was only identified in the dataset of differentially expressed genes at 12 hpi. This gene is similar to *WR3* in *A. thaliana* which is a wound responsive gene coding for nitrate transmembrane transporter. Three and 16 differentially expressed genes in the set of novel transcripts were also identified on scaffold 5 at 2 and 12 hpi, respectively (Table S4 and Table S5). However, none of those genes were in the confidence interval of the apricot QTL. This very low number of differentially expressed genes identified on scaffold 5 of peach in the confidence interval determined on linkage group 5 of apricot may reflect the fact that we focused on the identification of genes involved in early infection response in the peach genome whereas the QTL identified in apricot was based on disease severity data obtained 42 days post inoculation.

Sherif and colleagues [12] studied the level of expression of seven pathogenesis-related (PR) genes at 1, 4, 8, 24 and 48 hpi in the peach cultivar ‘Venture’ resistant to *X. campestris* (syn. *arboricola*) pv. *pruni* and in the susceptible cultivar ‘BabyGold 5′. In a second study from Sherif and colleagues [74], differentially expressed ethylene response factors (ERFs) after *X. arboricola* pv. *pruni* inoculation were identified in the same cultivars and at the same time points. To identify the transcripts corresponding to these genes on the peach genome, an analysis was performed using these accessions as BLASTN queries (GenBank, accessions nos. HQ825094 to HQ825098, and JF694923 to JF694927). A total of 23 genes potentially corresponding to the ERF and PR genes were found. From the 17 potential PR genes, 11 were not differentially expressed, five were not expressed at all and one was up-regulated only at 2 hpi, with a log_2_ fold change of 1.2 (Table S7). From the six potential ERF genes found, four were not differentially expressed, one was not expressed and only one was up-regulated at 2 hpi. These differences obtained between the studies could be due to the difference of inoculation technique used, explaining why diverse sets of genes may be involved in both studies. Indeed, in Sherif and colleagues experiments, plants were dipped into the inoculum, whereas here the inoculum was directly injected into the leaves and measurements were done at slightly different time points.

### Conclusions

RNA-seq technology is a very valuable tool to enhance our understanding of the genetics underlying the resistance mechanisms in pathosystems. This study is the first to give a global view of the gene expression of a *Prunus* crop under pathogen attack. We provide insights of the peach transcriptome once bacterial cells of *X. arboricola* pv. *pruni* have passed physical barriers and are inside the leaf. Because plants may recognize the bacterial effectors and induce a hypersensitive response within 24 h [75], we performed comparisons of inoculated samples with their respective controls at the early stages of 2 and 12 hpi. Although the peach variety used in our study is moderately susceptible, many genes with potential defense-related functions were differentially expressed at 12 hpi. Fewer genes were differentially expressed at 2 hpi, however the GO annotations and classifications showed that genes belonging to a wide range of functional categories were already involved in the defense response to the pathogen at this early time point. Furthermore, the RNA-seq technology not only permitted to identify differentially expressed genes involved in basal or gene-for-gene defense mechanisms, but also revealed novel differentially expressed genes and transcripts of unknown functions. Thus, this study provides an important basis for further characterization of peach defense-related genes in response to *X. arboricola* pv. *pruni* infection. The results obtained will be used to support further research on the pathogen transcriptome and characterize the host-pathogen molecular interactions.

## Supporting Information

Figure S1 **Number of genes expressed at different sampling depths. Genes with FPKM values obtained by Cufflinks v.2.0.1 higher than zero were considered as expressed.** Max is the total number of reads obtained in each sample.
(TIFF)

Table S1 **Complete list of differentially expressed peach genes at 2 h post inoculation with** ***X. arboricola*** **pv.** ***pruni.***
(XLSX)

Table S2 **Complete list of differentially expressed peach genes at 12 h post inoculation with** ***X. arboricola*** **pv.** ***pruni.***
(XLSX)

Table S3 **List of differentially expressed peach genes in common at 2 h and 12 h post inoculation with** ***X. arboricola*** **pv.** ***pruni.***
(XLSX)

Table S4 **Complete list of peach novel transcripts that are over or under represented at 2 h post inoculation with** ***X. arboricola*** **pv.** ***pruni.***
(XLSX)

Table S5 **Complete list of peach novel transcripts that are over or under represented at 12 h post inoculation with** ***X. arboricola*** **pv.** ***pruni.***
(XLSX)

Table S6 **List of peach novel transcripts that are over or under represented at both 2 h and 12 h post inoculation with** ***X. arboricola*** **pv.** ***pruni*** **.**
(XLSX)

Table S7 **List of putative transcripts that could correspond to the PR and ERF genes identified by Sherif and colleagues** [**12**] **** [**74**] **.**
(XLSX)
