# Supplementary figures and images for: Comparative RNA-Seq Analysis of Early-Infected Peach Leaves by the Invasive Phytopathogen Xanthomonas arboricola pv. pruni
Source: PLoS One. 2013 Jan 14;8(1):e54196. doi: 10.1371/journal.pone.0054196 (PMC3544827; doi:10.1371/journal.pone.0054196)

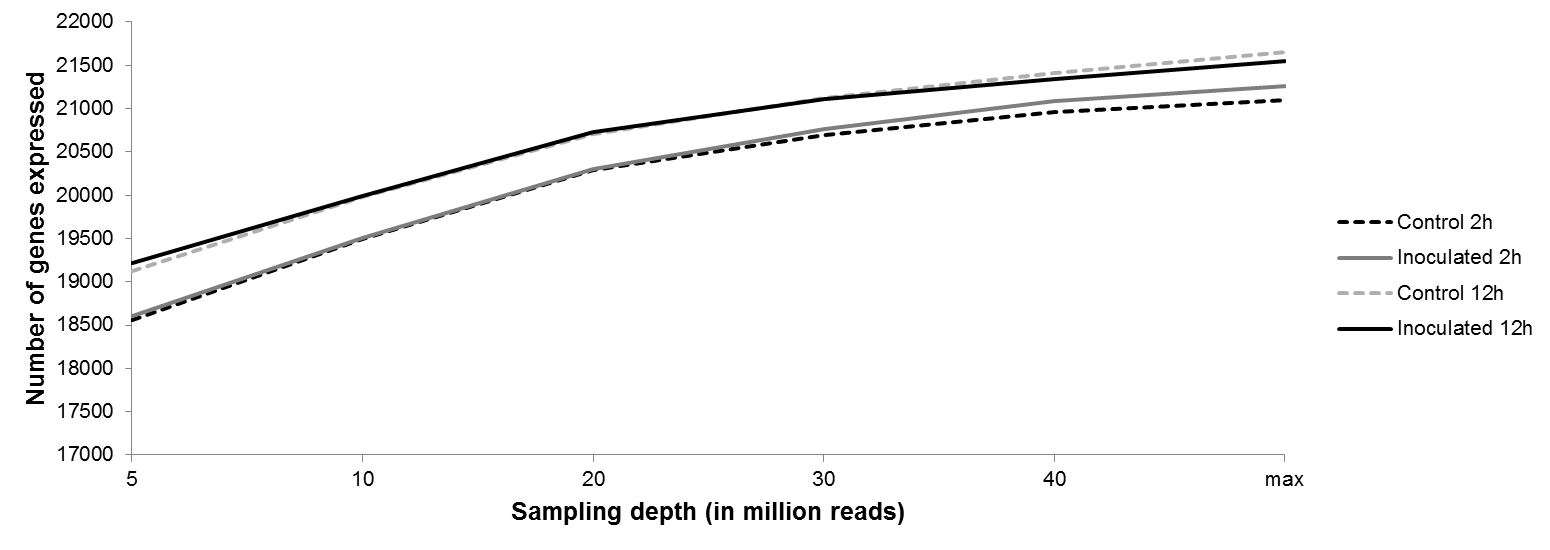

Supplement: Figure S1 — Number of genes expressed at different sampling depths. Genes with FPKM values obtained by Cufflinks v.2.0.1 higher than zero were considered as expressed. Max is the total number of reads obtained in each sample. (TIFF) [file pone.0054196.s001.tiff]
